# CRISPR-Based Diagnostics: A Potential Tool to Address the Diagnostic Challenges of Tuberculosis

**DOI:** 10.3390/pathogens11101211

**Published:** 2022-10-20

**Authors:** Yong Qi, Kun Li, Yuxi Li, Donglin Guo, Jing Xu, Yuexi Li, Wenping Gong

**Affiliations:** 1 Huadong Research Institute for Medicine and Biotechniques, Nanjing 210002, China; 2 Tuberculosis Prevention and Control Key Laboratory/Beijing Key Laboratory of New Techniques of Tuberculosis Diagnosis and Treatment, Senior Department of Tuberculosis, The Eighth Medical Center of PLA General Hospital, Beijing 100091, China

**Keywords:** tuberculosis, Xpert, CRISPR-based diagnostics, sensitivity, specificity

## Abstract

Tuberculosis (TB) is an infectious disease caused by *Mycobacterium tuberculosis*, which infects more than 23% of the world’s population. With the emergence of drug-resistant TB (DR-TB) and latent TB infection (LTBI), rapid diagnosis of DR-TB and LTBI has become a challenge for the prevention and control of TB. Herein, we highlight these challenges and discuss emerging clustered regularly interspaced short palindromic repeats (CRISPR)-based diagnostics in TB detection. Currently, the clinical diagnosis of *M. tuberculosis* infection mainly depends on pathogenic and molecular biological methods, including sputum smear, sputum culture, and Xpert. Although CRISPR-based diagnostics have not been applied to the clinical diagnosis of TB, they have shown exciting preponderances in TB diagnosis compared with traditional methods, including higher sensitivity, less sample input, and shorter turnaround time. CRISPR-based diagnostics represent a potential tool to address the challenges and natural weaknesses associated with traditional TB diagnosis methods. Based on the currently available data, we suggest that future CRISPR-based TB diagnostics should be developed in the direction of automation, modularization, diversification, and intelligence. By combining the CRISPR platform with various systems, such as microfluidic chips, droplet microfluidics, electrochemical techniques, and optical systems, the specificity and sensitivity of TB diagnosis may be revolutionized.

## 1. Introduction

Tuberculosis (TB) is an infectious disease caused by *Mycobacterium tuberculosis* (Mtb), which infects more than 23.0% of the world’s population and has become a global health problem [1]. It is also the second leading cause of death (only behind COVID-19) among infectious diseases, with mortality nearly double that of human immunodeficiency virus/acquired immune deficiency syndrome (HIV/AIDS) [2], with the first year-over-year increase in 2020 despite years of effort to end TB. The difficulty associated with TB diagnosis is believed to be one of the major obstacles to successful prevention and control of the disease [3]. In 2020, 41.4% to 47.3% of cases failed to be diagnosed and reported [2]. The bacteriological confirmation of TB is critical for correct diagnosis, guiding first-line and second-line anti-TB drug use, as well as effective and timely treatment, whereas more than 40% of globally confirmed pulmonary TB cases were not bacteriologically confirmed each year since 2005 [2]. In the present study, the current diagnostic challenges of TB are discussed, novel clustered regularly interspaced short palindromic repeats (CRISPR)-based diagnostics and their applications in TB diagnosis are reviewed, as well as their potential to resolve the diagnostic challenges.

## 2. Diagnostic Challenges of TB

The fast, sensitive, and accurate diagnosis of TB and drug-resistant (e.g., rifampicin, isoniazid, and pyrazinamide) TB (DR-TB) is crucial to reducing morbidity, mortality, and transmission among patients. However, enormous challenges are associated with this task. As the primary test for TB diagnosis, sputum smear microscopy cannot distinguish *Mycobacterium tuberculosis* (MTB) from nontuberculosis mycobacteria, with relatively low detection sensitivity, especially on sputum specimens with a low amount of MTB [4]. The culture-based mycobacterial detection method is the gold standard for TB diagnosis. It has high sensitivity, specificity, and accuracy. However, it requires professional operator and usually takes a long time (up to 4 to 6 weeks) to obtain results, preventing rapid treatment of patients. More than ten highly sensitive and specific nucleic acid amplification tests (NAATs) have been introduced to date and are recommended by the World Health Organization (WHO) for bacteriological confirmation of TB and DR-TB. Such tests have revolutionarily improved the TB diagnostic landscape by significantly lowering the limit of detection (LOD), enhancing sensitivity, and reducing the time needed for diagnosis compared with traditional culture testing or sputum smear microscopy. For instance, the GeneXpert MTB/RIF (Xpert) assay has substantially improved the diagnosis of TB and DR-TB [2] through full automation, integration, and improved accuracy.

However, it was reported that the Xpert test has not improved global detection rates substantially, showing limited efficacy in diagnosing extrapulmonary tuberculosis due to its inadequate sensitivity for some bacilli clinical specimens [5]. These paucibacillary specimens are usually from smear-negative pulmonary TB, extrapulmonary TB, pediatric TB, and HIV-positive TB patients, resulting in acid-fast bacillus (AFB)-smear negativity and representing one of the biggest challenges in TB diagnosis [6]. Furthermore, the high cost of the Xpert MTB/RIF system limits its usefulness in many high-TB-burden countries, leading to significant variation in bacteriological confirmation rates in pulmonary TB among countries, with lower levels of confirmation in low-income countries and higher levels in high-income countries [2]. Therefore, novel, cost-effective, and ultrasensitive methods capable of rapid screening and diagnosis of TB are urgently needed [7].

## 3. CRISPR-Based Diagnostics in TB Diagnosis

**Figure 1:**
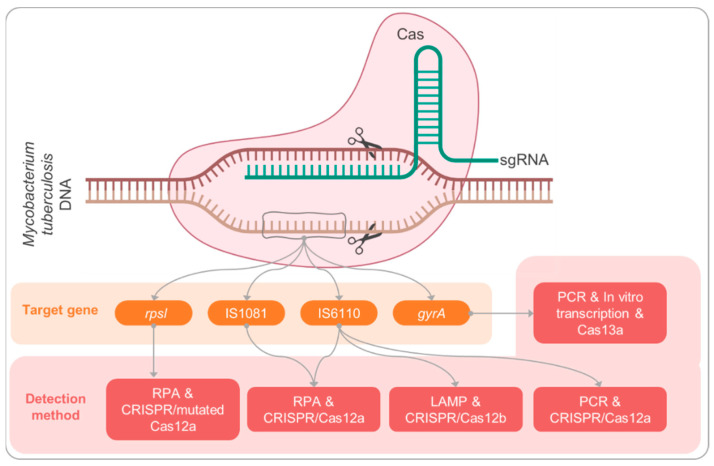
Schematic diagram of Mycobacterium tuberculosis diagnosis based on CRISPR technology.

The emerging CRISPR/CRISPR-associated (Cas) system is a gene-editing tool and a next-generation pathogen detection method that can detect single-nucleotide polymorphisms (SNPs) with high sensitivity and specificity. Cas protein and single guided RNA (sgRNA) form a complex of Cas/sgRNA to specifically recognize RNA (Cas13a and Cas13b) or DNA (Cas12a and Cas14) targets (Figure 1). They activate the *trans*-cleavage activity of Cas protein to degrade reporters for target detection. “SHERLOCK”, “DETECTR”, and “Holmes” systems, as well as a few other nucleic acid detection technologies using the CRISPR/Cas platform for pathogen detection, tumor diagnosis, and on-site detection, have emerged [8,9,10]. The fifth annual roundup of tools with the potential to shake up science published by *Nature* listed CRISPR-based diagnostics as one of the seven technologies to watch in 2022 [11]. Combined with nucleic acid amplification strategies, CRISPR/Cas platforms can rapidly, accurately, and cost-effectively detect nucleic acid at attomolar concentration levels, promising to address the diagnostic challenges of TB. In the present study, we collected and reviewed related literature on the topic of diagnosis of TB using CRISPR/Cas-based method in the PubMed database (https://pubmed.ncbi.nlm.nih.gov/ (accessed on 25 August 2022)) using searching keywords “CRISPR”, “Tuberculosis”, and “detection”. Reviews and unrelated publications were excluded, and the remaining publications were reviewed.

CRISPR-based diagnostics for MTB detection have yielded superior results relative to conventional methods (Table 1). Diagnosing clinically paucibacillary TB patients, especially those with AFB-smear negativity, is one of the biggest challenges in TB diagnosis. Sam et al. combined loop-mediated isothermal amplification (LAMP) with CRISPR/Cas12b detection to develop a novel MTB DNA detection platform (TB-QUICK) [12]. The LOD of the assay reached as low as 1.3 copies/μL without cross reacting to clinically prevalent nontuberculous mycobacteria strains. The TB-QUICK assay showed comparable sensitivity in detecting AFB-positive TB patients with MTB culture and Xpert assays (100%, 100%, and 95.8%, respectively), with dramatically higher sensitivity in detecting AFB-negative TB patients, making it more advantageous for the diagnosis of paucibacillary TB patients. A similar study targeting the MTB complex was conducted by coupling LAMP with CRISPR/Cas12a, using the end-point detection of either fluorescent detection or lateral flow test [13]. The established method, with an LOD of about 10 copies/reaction and a specificity of 100%, performed better than conventional culture, AFB smear test, and Xpert MTB/FIR in detection of sputum samples with a low MTB complex pathogen load.

A well-known weakness of the conventional method lies in diagnosing extrapulmonary TB [6]. Ai et al., for the first time, combined a recombinase polymerase amplification (RPA) assay with Cas12a-based detection (CRISPR-MTB) to develop a rapid and highly sensitive method for both pulmonary and extrapulmonary TB diagnosis [5]. The CRISPR-MTB, with an overall improved sensitivity (79%), less required sample input (500 μL), and shorter turnaround time (1.5 h) compared with both culture and Xpert, as well as high specificity (98%), demonstrated considerable potential as a new diagnostic method for TB, especially for extrapulmonary TB [7]. Another similar study targeting the IS1081 gene, although with a higher LOD (4.48 fM or 298 copies/reaction vs. 12.5 copies/reaction), demonstrated excellent sensitivity (99.29%) and specificity (100%) in the detection of samples of pulmonary TB [4]. However, its potential for detection of extrapulmonary MTB was not evaluated.

Typically, detection of circulating MTB-derived cell-free DNA (cfDNA) instead of sputum samples has the potential to mitigate underdiagnosis in individuals with extrapulmonary TB. Sensitivity tests for cfDNA benefit diagnosis and monitoring of the treatment effect. However, conventional PCR-based detection has performed poorly, with highly variable diagnostic sensitivity in the detection of MTB cfDNA [14]. Additionally, the above-mentioned TB-QUICK assay did not perform well in detecting MTB cfDNA. Recently, Huang et al. established an ultrasensitive PCR-CRISPR-mediated assay with an LOD of 0.25 copies/µL. Its sensitivity and specificity were 96% and 94% in an HIV-negative adult cohort, 83% and 95% in a pediatric cohort, and 100% and 85% in a people living with HIV (PLHIV) cohort, respectively [14]. By optimizing both cfDNA extraction and amplification procedures, the assay could sensitively detect serum MTB cfDNA in most patients with TB, especially paucibacillary patients missed by sputum-based diagnostics. Furthermore, the authors introduced an RPA assay and lateral flow test to achieve visual detection, which could be applied in a portable format suitable for resource-limited settings. The findings proved the utility of CRISPR-based diagnostics in serum MTB cfDNA analysis to provide non-sputum-based TB diagnosis.

CRISPR-based diagnostics have also been developed for detection of mutant drug-resistant genes or single-base mutation of MTB, such as streptomycin- and fluoroquinolone-resistant mutations, demonstrating the potential use for SNP detection and early drug resistance monitoring, thus guiding clinical treatment [15,16,17].

## 4. Limitations of CRISPR-Based TB Diagnostics

Because CRISPR-based diagnostics have only recently been developed (less than 5 years), potential challenges are still encountered with respect to rapid translation of laboratory results into practice. Typically, CRISPR-based diagnostics require an amplification procedure to ensure high sensitivity, making detection complicated and time-consuming and introducing risks of amplification bias and cross contamination. Introducing one-pot procedures or using amplification-free strategies a key for practice use in the future [18]. Additionally, the studies reviewed herein used only one target gene to detect MTB, which might be absent in some MTB strains, resulting in false-negative results. In this respect, multiplex detection methods targeting different genes need to be developed [14]. Moreover, compared with the frequently used real-time fluorescent PCR, aspects of quantitative detection, automation, and high-throughput use still need to be improved for CRISPR-based TB diagnostics.

## 5. Prospects and Conclusions

Overall, CRISPR-based diagnostics exhibit improved diagnostic performance relative to traditional methods, such as culture and Xpert, with higher sensitivity, lower sample input, and shorter turnaround time [7], indicating their potential as a tool to address diagnostic challenges and weaknesses associated with conventional methods for MTB testing, such as extrapulmonary TB, pediatric TB, HIV-positive TB, and other paucibacillary TB. Based on the reviewed reports, we suggest that future CRISPR-based TB diagnostics should be developed toward automation with integrated nucleic acid extraction procedures, high-throughput, a multiplex of drug-resistance genes, absolute quantitation, and nucleic acid amplification-free by a combination of CRISPR platform with various systems, such as microfluidic chips, droplet microfluidics, electrochemical techniques, optical systems, etc. [19].

Although CRISPR-based diagnostics have not been applied for clinical diagnosis of TB, the CRISPR-based high-throughput COVID-19 test developed by Mammoth Biosciences has received FDA emergency use authorization, indicating broad application prospects of CRISPR-based diagnostics for TB diagnosis.

## Figures and Tables

**Table 1:** CRISPR-based diagnostics for Mycobacterium tuberculosis (MTB) detection.

No.	Detection Method	Target Gene	Detection Limit	Sensitivity	Specificity	Advantage
1	RPA and CRISPR/Cas12a (fluorescent detection)	IS6110	5 copies/µL (12.5 copies/reaction) or 50 CFU/mL	79% (91/116)	98% (62/63)	Overall higher sensitivity and shorter detection time than both Xpert and culture methods [7].
2	RPA and CRISPR/Cas12a (fluorescent detection)	IS1081	4.48 fmol/L (about 298 copies/reaction)	99.29% (139/140)	100% (53/53)	Specificity, sensitivity, and accuracy comparable with the result of the gold-standard culture method [4].
3	LAMP and CRISPR/Cas12b (fluorescent detection)	IS6110	1.3 copies/µL (2.6 copies/reaction)	86.8% (59/68)	95.2% (20/21)	Extra-high sensitivity and specificity in TB pulmonary samples with AFB negativity, making it advantageous for diagnosis of paucibacillary TB patients [12].
4	LAMP and CRISPR/Cas12a (fluorescent detection or lateral flow test)	IS6110	About 10 copies/reaction	79.5% (35/44)	100% (no cross reactions to non-MTB complex strains)	Higher detection rate than conventional culture, AFB smear test, and Xpert MTB/FIR in detection of sputum samples with a low MTB complex pathogen load [13].
5	(1) PCR and CRISPR/Cas12a (fluorescent detection); (2) RPA and CRISPR/Cas12a and lateral flow test (visual detection)	IS6110	0.25 copies/μL of purified cfDNA or 0.06 copies/μL of serum sample	96% (27/28 HIV-negative adults); 83% (5/6 pediatric TB); 100% (13/13 HIV-positive TB patients)	94% (16/17 HIV-negative adults); 95% (21/22 children); 85% (39/46 HIV-positive people)	Requirement of cfDNA isolated from 200 μL of serum, with a 2 h sample-to-answer time and suitable for paucibacillary specimen screening in resource-limited settings [14].
6	RPA and CRISPR/mutated Cas12a (fluorescent or visual detection)	Gene locus mutations in the rpsL gene	0.05 ng MTB whole genome; distinguishing of mutation rates of 0.1%	100%	100%	The entire detection process can be completed within 60 min; an alternative for detecting streptomycin-resistant TB [15].
7	PCR, in vitro transcription, and Cas13a (fluorescent detection)	Partial sequence of the quinolone resistance-determining region in the gyrA gene	Distinguishing Of mutations from wild-type samples varied from 1 × 10^0^ to 1 × 10^2^ copies/mL	91.4%	100%	Potential for use in single-nucleotide polymorphism detection in quinolone-resistance gene mutations; crRNA screening strategy useful for early drug resistance monitoring and guidance for clinical treatment [16].
8	Hybridization chain reaction (HCR) and Cas9	16S rRNA	30 CFU/mL of MTB strain H37Ra	88.9% (24/27)	95.6% (22/23)	The method was simple, rapid, and accurate, with the potential to realize single-base mismatch detection [17].

## Data Availability

Not applicable.
